# FT-IR study of the polysaccharides isolated from the skin juice, gel juice, and flower of *Aloe vera* tissues affected by fertilizer treatment

**DOI:** 10.1186/2191-2858-2-33

**Published:** 2012-10-24

**Authors:** Fatemeh Nejatzadeh-Barandozi, Sattar Tahmasebi Enferadi

**Affiliations:** 1 Department of Horticulture, Faculty of Agriculture, Khoy Branch, Islamic Azad University, P.O. Box 58168–44799, Khoy, Iran; 2 National Institute of Genetic Engineering and Biotechnology (NIGEB), P.O. Box 14155–6343, Tehran, Iran

**Keywords:** Aloe vera, Polysaccharides, Skin juice, Gel juice, Flower, Fertilizer

## Abstract

**Background:**

This experiment was conducted to evaluate the effect of different amounts of fertilizers on the polysaccharides of *Aloe vera* plant. There were four different treatments, viz*.* T_1_ = 150% N, T_2_ = 150% P, T_3_ = 150% K, and T_4_ = 150% NPK (50% N + 50% P + 50% K) soil. Crude water-soluble polysaccharides were isolated from the gel juice, skin juice, and flowers of *A. vera* planted in these soils.

**Results:**

Result indicates that skin juice contained 2.4 times the level of polysaccharides in gel juice from one plant, suggesting the potential industrial application of *A. vera* skin rather than discarding it. After anion-exchange chromatography, neutral polysaccharides accounted for 58.1% and 78.5% of the total recovered neutral and acidic polysaccharide preparations from the gel juice and skin juice, respectively, whereas the crude flower polysaccharides were largely composed of weakly acidic polysaccharides (84.2%). Sugar analysis of the polysaccharides after gel permeation chromatography revealed that glucose and galactose were the most abundant monosaccharide in the neutral polysaccharides from the gel juice and skin juice, respectively. The acidic polysaccharides from the two juices consisted of glucuronic acid, galactose, glucose, mannose, and xylose with variable proportions.

**Conclusions:**

Except glucuronic acid (15.4%) in flower acidic polysaccharide, the flower neutral and acidic polysaccharides contained galactose, glucose, and mannose as the main sugar components. Glucuronic acid was the major uronic acid in all acidic polysaccharides from different tissues.

## Background

*Aloe vera* (Liliaceae), one of the most widely cultivated species of the genus *Aloe* in the world, has been widely used for medicines and cosmetics, and its chemical constituents have been studied [1-4], different properties being ascribed to the inner, colorless leaf gel and to the exudate produced by the bundle sheath cells on the outer margin of the leaf. These major active ingredients, acting alone or in concert, include polysaccharides, glycoproteins, infiltrating exudate phenolics, and even, simplest of all, water. Polysaccharide fractions from water extracts of whole leaves of *A. vera* were found to lower blood glucose levels in normal mice and in alloxan-induced diabetic mice [5,6].

Several workers have tried the separation of *A. vera* gel carbohydrate polymers into their polysaccharide components [5,7,8]. However, the types and molecular sizes of the polysaccharides extracted from aloe gel appear very diverse. The controversy of the previous reports may be due to plant subspecies or different geographical origin, seasonal and cultivar variation, technical differences used to isolate the polysaccharide, or degradation of polysaccharides by endogenous enzyme activity [9-11].

Now, the processing of *A. vera* gel, derived from the leaf pulp of the plant, has become a big industry worldwide due to its application in pharmaceutical and cosmetic industries. However, more and more aloe skin was discarded as waste. Very little is known about the bioactive substances in *A. vera* skin and the potential use of aloe skin in industry. The leaf skin preparation from *A. vera* was shown to lower blood glucose, and in artificially induced diabetic animals, normal insulin production was resumed [12]. In yet another study, a carboxypeptidase was prepared from the ‘leaf skin’ and partially purified [3,10,13]. The compositional features of skin polysaccharides, alcohol insoluble complex, from other aloe species (*A. vera*) skin tissue have been studied [11]. In addition, it has been reported that the flower of *A. vera* was rich in ascorbic acid [4,14] and volatile components [6]. To our knowledge, however, the isolation, purification, and compositional analysis of polysaccharides from the skin juice and flowers of *A. vera* effected by fertilizers have not been performed yet. In the present study, we report the fractionation and chemical composition of polysaccharides isolated from the skin juice and flowers of *A. vera* effected by fertilizers*.* The comparison of the chemical features of these polysaccharides with gel juice polysaccharides is also taken into consideration.

## Methods

### Plant material and tissue separation

This investigation was conducted at the National Institute of Genetic Engineering and Biotechnology (NIGEB), Tehran, Iran, during 2010. The climate of this area is subtropical. The soil of the experimental site was clay loam with a pH of 6.5. The experiment was laid out by randomized completely block design with four replications comprising four different treatments, viz*.* T_1_ = 150% N (100% N +50% P + 0% K), T_2_ = 150% P (50% N + 100% P + 0% K), T_3_ = 150% K (25% N + 25% P + 100% K), and T_4_ = 150% NPK (50% N + 50% P + 50% K) soil. Plants were grown in the greenhouse. Distance between the plants was 20 cm. Chemical fertilizers were used in this experiment as per treatment [5]. *A. vera* plant and dried *A. vera* flower were available for this investigation. Fresh whole leaves, between 25 and 45 cm in length corresponding to 3-year-old plant, were washed, and the spikes, placed along their margins, were removed before slicing the leaf to separate the epidermis or skin from the filet. The skin and filet were washed extensively with distilled water to remove the exudates from their surfaces. Further, the skin and filet were cut into cubes, blended in a food processor (model HC380D, Tefal, Rumilly, France) at low speed, and squeezed through a 200-mesh screen. *A. vera* gel juice and skin juice were then centrifuged at 5,200×*g* (Universal Centrifuge DL-5, Shanghai, China) for 10 min to discard the callus. *A. vera* flower was initially dried at 70°C in an oven and ground using a food processor. These dried powders (100 g) were suspended in 500 ml of distilled water and refluxed for 2 h at 80°C. This extraction procedure was repeated three times, and the supernatants obtained were pooled and filtered on a Whatman no. 2 paper to remove the insoluble materials. Then, the clear supernatant was concentrated in a rotary evaporator under reduced pressure.

### Extraction of crude polysaccharides from aloe skin juice, gel juice, and flower

The skin juice, gel juice, and the extracted flower solution, respectively, was mixed with four times volume of 95% (*v*/*v*) ethanol, stirred vigorously, and left overnight at 4°C. The precipitate was centrifuged at 5,200×*g* for 10 min, discarding the supernatant. The precipitate was redissolved in distilled water overnight and precipitated again by addition of four volumes of ethanol. The obtained precipitate was suspended in distilled water and treated with Sevag reagent (1-butanol:chloroform 1:4, *v*/*v*); after vigorous stirring on a vortex mixer for 10 min at room temperature and centrifugation at 5,200×*g* for 10 min, the supernatant solution was retreated in this manner until there was no free protein [15]. Then, the flower polysaccharide solution was further treated with 2% activated charcoal to decolorize. The resulting solution was added with three volumes of 95% (*v*/*v*) ethanol to precipitate the polysaccharides. The crude polysaccharides were washed two times with absolute ethanol, acetone, and ethylether, and then completely dried at 40°C in an air oven for 48 h. The weights of the crude polysaccharides from the gel juice, skin juice, and flower were estimated respectively.

### FT-IR spectroscopy

The crude polysaccharides were characterized using a Fourier transform infrared spectrophotometer (FT-IR; FTIR 8400S, Shimadzu, Tokyo, Japan). The dried polysaccharides were ground with KBr powder and pressed into pellets for FT-IR spectra measurement in the frequency range of 400 to 4,000 cm^−1^.

### Purification of crude polysaccharides

The crude polysaccharides were separated by anion-exchange chromatography on a DEAE-Sephadex A-25 column (26 cm × 3 cm; GE Healthcare, Fairview, CT, USA). An amount of 50 mg of crude gel polysaccharide (CGP), crude skin polysaccharide (CSP), or crude flower polysaccharide (CFP) was dissolved in 10 ml of distilled water, filtered through a Millipore 0.45-μm filter (Millipore Co., Billerica, MA, USA), and applied onto the column at a flow rate of 1.2 ml/min. The neutral fraction was eluted with distilled water, while the weakly acidic fraction was eluted with 0.5 M NaCl, and then, the strongly acidic fraction was eluted with 1.0 M NaCl. Carbohydrates were detected in the 5-ml fractions by the phenol-H_2_SO_4_ method [16]. The fractions with significant amount of carbohydrates were pooled, concentrated, dialyzed, and freeze-dried.

The freeze-dried polysaccharides were further purified, and the molecular weight distribution of the samples was measured by gel permeation chromatography on a Sepharose 4B column (42 cm × 2 cm, GE Healthcare). The column was calibrated with T-series dextrans of known molecular weights. The eluent was 0.1 M NaCl at a flow rate of 18 ml/h, and aliquots of 3 ml were collected. The fractions were assayed for carbohydrate as described above.

### Chemical analysis of the polysaccharides

The total carbohydrate content was determined by the phenol-H_2_SO_4_ method [16], using glucose as the standard. Purified polysaccharide preparations (10 mg) were hydrolyzed with 2 M H_2_SO_4_ (5 ml, 100°C, 8 h) in a sealed ampoule under a nitrogen atmosphere [16], and the residual acid was neutralized with BaCO_3_ at 45°C. After centrifugation (5,200×*g*, 10 min), the supernatants were filtered through a Millipore 0.45-μm filter. The TLC analyses of hydrolysates were performed on silica gel 60 G (Merck, North Ryde, Australia) plates with n-propanol/water/pyridine (17:3:1, *v*/*v*) as the developing phase, and the neutral monosaccharide(s) and uronic acids in the hydrolysate were detected by spraying with diphenylamine/aniline/phosphoric acid (5:5:1, *v*/*v*), dried, and heated at 80°C for 15 min. Authentic monosaccharide standards were run along with the test samples.

The percentages of different neutral sugars were estimated by gas–liquid chromatography (GC-14A, Simadzu Corporation, Kyoto, Japan) of derived alditol acetates using inositol as internal standard [17]. The column, SP-2330 fused silica (30 m × 0.32 mm × 0.25 mm), was maintained at 120°C for 2 min and then raised to 220°C at 25°C/min. N_2_ was used as the carrier gas (1 ml/min) with an injection volume of 1 ml. The concentrations of uronic acids in the hydrolysates were determined by the m-hydroxydiphenyl sulfuric acid method with a galacturonic acid standard [17].

## Results and discussion

### Preparation of the crude polysaccharides from skin juice, gel juice, and flower

**Figure 1:**
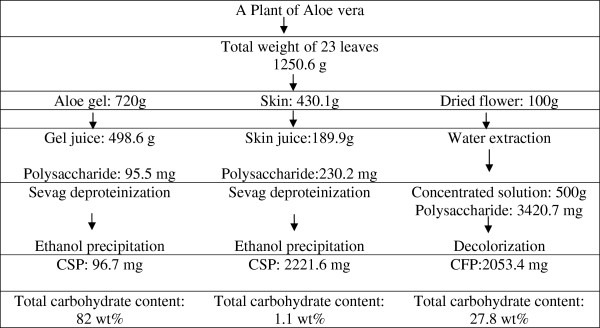
**Recovery process of crude polysaccharides from gel juice, skin juice, and flower of *A. vera.***

The CSP, CGP, and CFP were obtained from the skin juice, gel juice, and dried flower of *A*. *vera* according to the scheme in Figure 1. As seen from Figure 1, the skin tissue and gel tissue represented 34.4 g/100 g and 57.6 g/100 g entire fresh leaves of *A. vera*, respectively. After the preparation of skin juice and gel juice from the different tissues, the amount of polysaccharide in skin juice is 2.4 times that in gel juice. Although the yield of CSP from one whole plant was much higher (22-fold) than that of CGP, the total amount of carbohydrate detected in CSP fraction was 2.3-fold lower than that in CGP due to the lower percentage of polysaccharide in CSP (1.1%) compared to that in CGP (83.1%), while in CFP, this percentage was 28.7%. After water extraction, the flower polysaccharides in the aqueous solution amounted to 3.52% of the starting flower polysaccharides by dry weight, which is higher than that in other plant flowers (*Bombax malabaricum*, 1.54%) [18]. Nevertheless, during the preparation of CFP compared with CGP, the exhaustive extraction steps including deproteinization and decolorization are necessary due to the more complex composition in the flower as it was more contaminated with protein and colored materials or phenolic compounds. Especially, Sevag method had been repeated 25 times to remove protein, which led to the loss of more than 60% of the polysaccharide content, and the purity of CFP was still low even after extensive pretreatment steps. Furthermore, concentration of the extracted solution under reduced pressure and repeated centrifugation are the additional procedures for the preparation of CFP.

**Figure 2:**
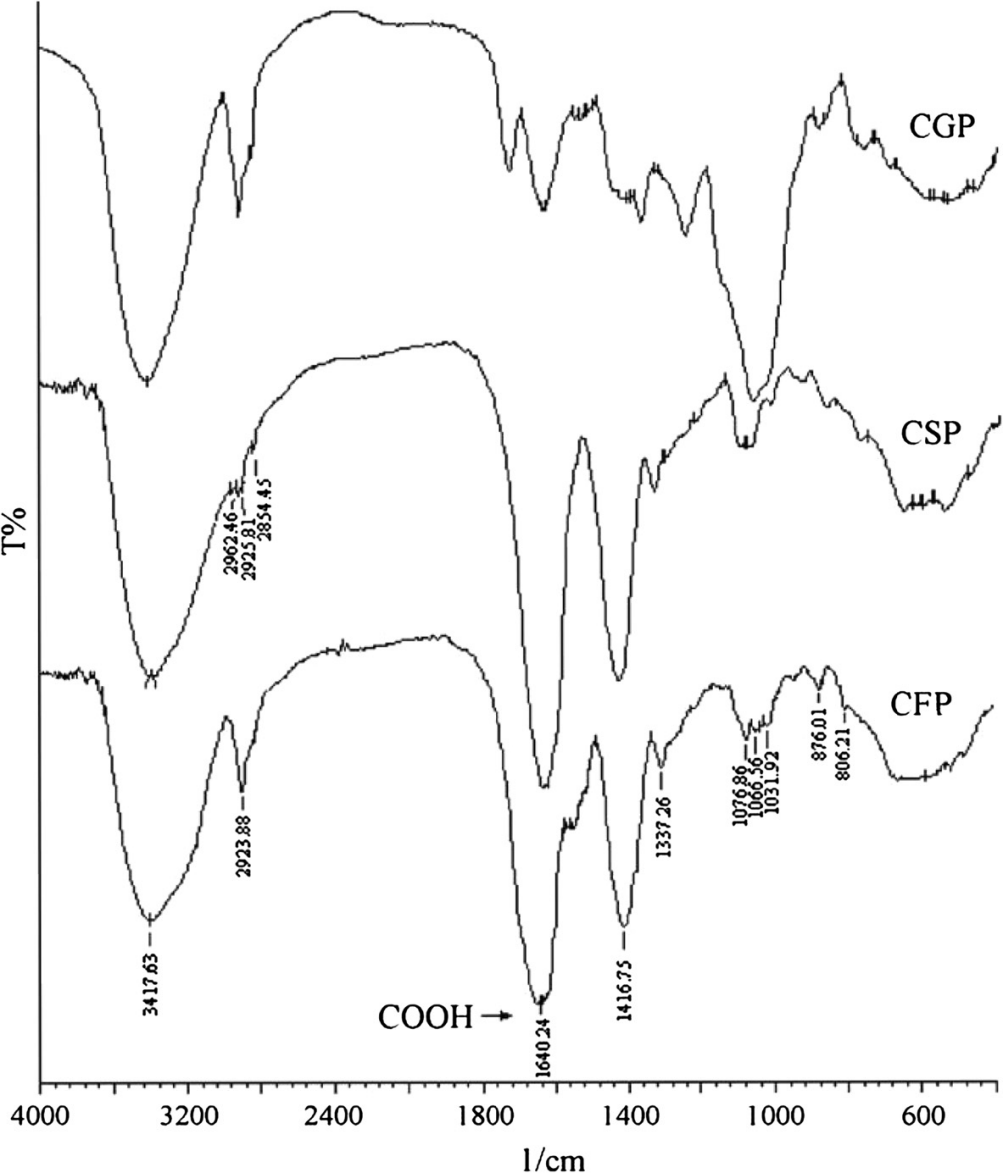
**Infrared spectra of the crude polysaccharides from *A. vera *gel juice, skin juice, and flower.**

Protein and nucleic acids were not detected in the crude polysaccharides by UV spectrometry. Figure 2 showed the infrared spectra of CGP, CSP, and CFP. In Figure 2, the hydroxyl group and ether signals (C-O-C) in sugar units were absorbed as strong intensities around 3,420 and 1,070 cm^−1^, respectively. The band at 1,066 cm^−1^ in gel polysaccharides arises from the mannopyranose component [19]; the weak bands in CSP and CFP at this range indicated a small amount of mannose present in skin polysaccharide and flower polysaccharide. A further band at 1,031 cm^−1^ was due to the glucan units. The characteristic pyranoside ring absorption band at 876 cm^−1^ (C-H ring vibration) and mannose absorption peak at 806 cm^−1^ were detected in CGP, CSP, and CFP, and as already reported in gel polysaccharide from *A. vera* by Yan et al. [4]. Specifically, the peaks at 1,740 and 1,250 cm^−1^ ascertain the presence of o-acetyl ester. The highest amount of o-acetyl ester units in gel polysaccharide is reflected in the spectrum by the intense o-acetyl ester-related maximum. It was suggested that the presence of acetyl groups is necessary for biological activation, possibly because they cover a number of hydrophilic hydroxyl groups and thus make the molecule more able to cross hydrophobic barriers in the cell [10]. Meanwhile, the band at 1,740 and 1,250 cm^−1^ disappeared in skin polysaccharide and flower polysaccharide, which may indicate either that they were covered by the strong peak at 1,640 cm^−1^ or the absence of o-acetyl group in skin and flower polysaccharides. Further, the absorption bands of carboxyl groups at around 1,640 cm^−1^ (asymmetrical COO^−^ stretching vibration) and 1,417 cm^−1^ (symmetrical COO^−^ stretching vibration) are most pronounced in the FT-IR spectrum of skin polysaccharide and flower polysaccharide, which is in accord with the high carboxyl group content of the CSP and CFP fractions [20]. Furthermore, crystalline grain appeared in the sulfuric acid hydrolysate of CSP, probably due to the presence of oxalate insoluble residue co-precipitated with polysaccharides by adding ethanol to the skin juice, corresponding to the large amount of calcium oxalate crystalline substances located within the cells of the green skin tissue [21]. Strong IR absorption bands are also present in the CSP and CFP spectra at 1,077 cm^−1^ and assigned to the higher amount of galactose units in CSP and CFP [19].

### Purification and composition of polysaccharides

Table 1 shows the yield, molecular weight distribution, and monosaccharide composition of the fractions. SN was separated by gel permeation chromatography (GPC) into a single fraction, termed SN1, with the molecular weight distribution from 2 to 59 kDa with a peak at 12 kDa and yield of 0.48% of CSP, which was not reported previously. Meanwhile, the GPC chromatography of GN yielded two fractions, GN1 and GN2; the molecular weight distribution was from 230 to 3,340 kDa with a peak at 690 kDa for GN1 and 115 to 207 kDa with a peak at 171 kDa for GN2, and yields of 29.2% and 5.1% of CGP, respectively. The two neutral fractions from gel juice (GN1 and GN2) shared a very similar composition pattern marked by the overwhelming proportion of glucose (about 80% of glucose glycosyl residues and 14% mannose residues, a little amount of galactose 3.2% to 5.1%), but appeared to be different in their molecular weight distribution. However, the neutral polysaccharide from skin juice (SN1) exhibited very different carbohydrate composition from those of gel juice. In the skin fraction SN1, galactose was the main type of monosaccharide, accounting for 62.8% of the total carbohydrate, followed by glucose for 29.7%; the amount of mannose detected represented only 6.2% of the total sugars. The previous reports on the types and molecular weights of the polysaccharides extracted from aloe gel appear very diverse. Wozniewski et al. separated the gel polysaccharides of *A. vera* into two partially acetylated glucomannans with molecular weights of 1,000 and 12 kDa, respectively, but in a same glucose-to-mannose ratio of 5:95, and one arabinogalactan containing arabinose, galactose, rhamnose, and glucose in a ratio of 43:43:7:7 with a molecular weight of 50 kDa [7]. Hikino et al. found mannoglucan (Man/Glc = 0.3:1, 57 kDa) and acetylated glucorhamnogalactan (Glc/Rha/Ga = 0.3:0.3:1, 12 kDa) [5]. According to Yagi et al. [8], glucan, arabinogalactan (Arab/Gal = 1.5:1) and acetylated mannan (Man/Ac = 9:1) were the main components of *A*. *vera* filet with molecular weights of 15, 30, and 40 kDa, respectively. The difference of the molecular weights and chemical compositions detected in this test from the above previous reports on *A. vera* gel polysaccharides was probably due to various factors such as the preparation or isolation method of aloe gel and the source and cultivar conditions of *A. vera* plant. Particularly, our work attributed the discrepancies over the polysaccharide composition of different *A. vera* tissues to chemical purification steps. The GPC chromatography of FN yielded one fraction, designated FN1, with molecular weight distribution from 21 to 108 kDa with a peak at 56 kDa. From Table 1, it is obvious that the composition of FN1 is quite different from those of skin juice and gel juice neutral polysaccharides. FN1 fraction was composed mainly of glucose (40.3%), galactose (30%), mannose (22.9%), and small amount of xylose (3.1%) and rhamnose (2.0%). SA was separated by GPC into three acidic fractions, SA1, SA2, and SA3; the molecular weight distribution was from 440 to 5,575 kDa with a peak at 1,925 kDa for SA1, 90 to 290 kDa with a peak at 156 kDa for SA2, and 9 to 52 kDa with a peak at 31 kDa for SA3, which yielded 0.06%, 0.02%, and 0.02% of CSP, respectively. Due to the lesser recovery yields of SA2 and SA3 from the GPC, the composition analysis was only performed on SA1. As depicted in Table 1, SA1 was rich in glucuronic acid (30.2%); glucose and mannose were the major neutral sugars accounting for 27% and 21.7%, respectively, of the total carbohydrate. In addition, the significant amounts of galactose (10.90%) and xylose (8.1%) were also detected in SA1. Similarly, the GPC chromatography of GA yielded two acidic fractions, GA1 and GA2, with distinctly lower molecular weights compared with those of gel neutral polysaccharides (GN1 or GN2), from 15 to 110 kDa with a peak at 40 kDa for GA1 and 5 to 13 kDa with a peak at 9 kDa for GA2. GA1 was selected for composition analysis due to the relatively high recovery yield of CGP (17.7% for GA1, only 3.8% for GA2). GA1 mainly constituted galactose (36.5%), glucose (30.1%), and glucuronic acid (27.5%). A trace of mannose (2.0%) and xylose (1.2%) were also found in GA1. The GPC chromatography of FA gave two fractions, designated FA1 and FA2, the molecular weight distribution was 45 to 125 kDa with a peak at 73 kDa for FA1 and 5 to 30 kDa with a peak at 21 kDa for FA2. Except for a significant content of glucuronic acid (16.1%), FA1 had high amounts of galactose (27.8%) and glucose (20.1%), with smaller but significant amounts of mannose (14.3%), rhamnose (11.5%), and some xylose (7.9%). The higher amount of galactose units in flower polysaccharides is evidenced also by the intense galactose-related band at 1,077 cm^−1^. Totally, glucuronic acid was the major uronic acid, and galacturonic acid was not distinguishable in all the acidic polysaccharide fractions.

**Table 1:** **Yield, molecular weight distribution, and sugar composition (mol%) of *A. vera *polysaccharides**

	**Gel juice polysaccharides**				**Skin juice polysaccharides**				**Flower polysaccharides**		
	**GN1**	**GN2**	**GA1**	**GA2**	**SN1**	**SA1**	**SA2**	**SA3**	**FN1**	**FA1**	**FA2**
Yield (%)^a^	29.2	5.1	17.6	3.7	0.48	0.06	0.02	0.02	2.65	0.59	13.2
Molecular weight distribution (kDa)	230 to 3,340	115 to 207	15 to 110	5 to 13	2 to 59	440-5,575	90 to 290	9 to 52	21 to 108	45 to 125	5 to 30
Molecular weight of each peak (kDa)	690	171	40	9	12	1925	156	31	56	73	21
Sugar composition^b^											
Rhamnose	-	-	-	ND	-	-	ND	ND	2.0	ND	11.5
Xylose	-	-	1.2	ND	-	8.1	ND	ND	3.1	ND	7.6
Mannose	15.1	13.5	2.0	ND	6.2	21.7	ND	ND	22.9	ND	14.3
Glucose	80	81.2	30.1	ND	29.7	27.0	ND	ND	40.3	ND	20.1
Galactose	3.2	5.1	36.5	ND	62.8	10.90	ND	ND	30	ND	27.8
Glucuronic acid^c^	-	-	27.5	ND	-	30.2	ND	ND	-	ND	16.1

Yield, molecular weight distribution and sugar composition (mol%) of the polysaccharides from the skin juice, gel juice, and flower of *A. vera* purified after gel permeation chromatography. Hyphen denotes values that were not detected. ND, not determined. ^a^Percentage weight of crude polysaccharides recovered. ^b^Percentage mol of total carbohydrate content. ^c^Polysaccharide was hydrolyzed in 2 M sulfuric acid at 100 °C for 8 h. Absorbance at 530 nm was measured according to the m-hydroxydiphenyl method, and the amount was calculated as galacturonic acid.

**Figure 3:**
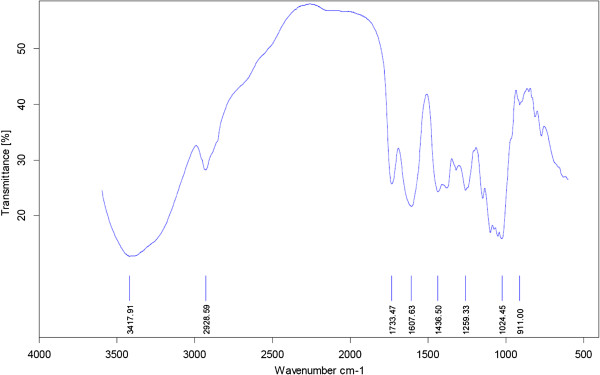
**The FT-IR spectra of the crude polysaccharides of *A. vera *in T150 N fertilizer treatment.**

**Figure 4:**
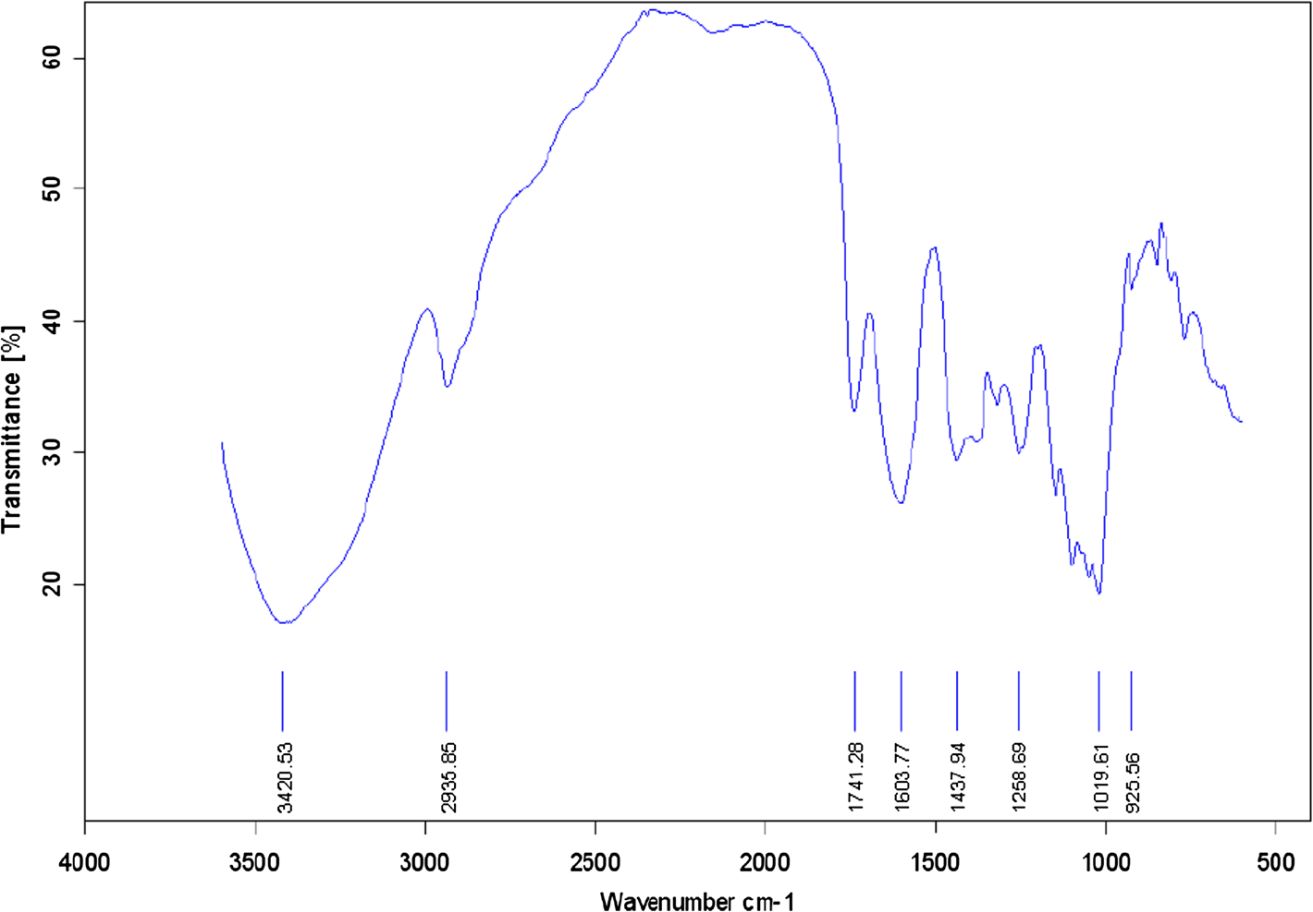
**The FT-IR spectra of the crude polysaccharides of *A. vera *in T150 P fertilizer treatment.**

**Figure 5:**
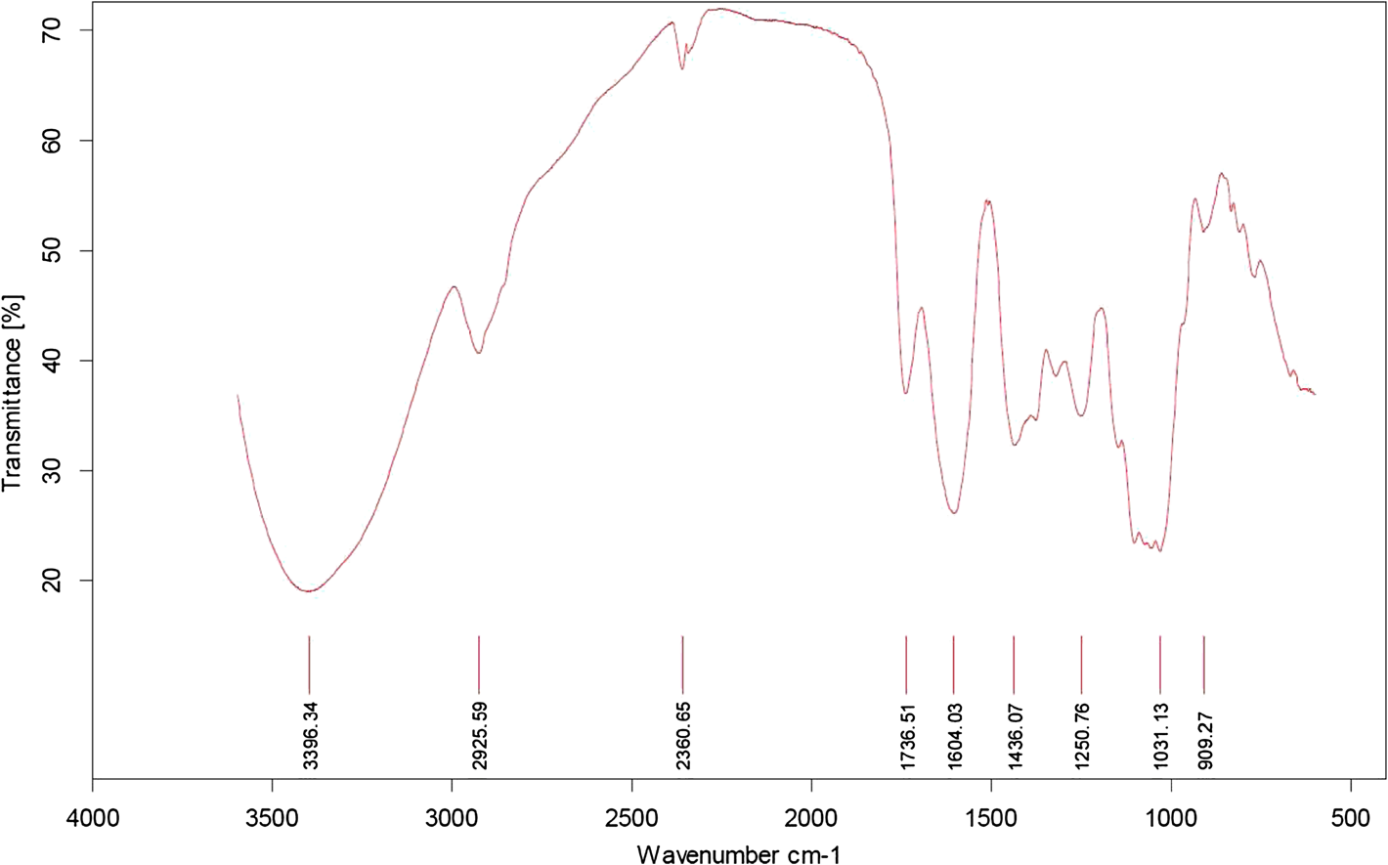
**The FT-IR spectra of the crude polysaccharides of *A. vera *in T150 K fertilizer treatment.**

**Figure 6:**
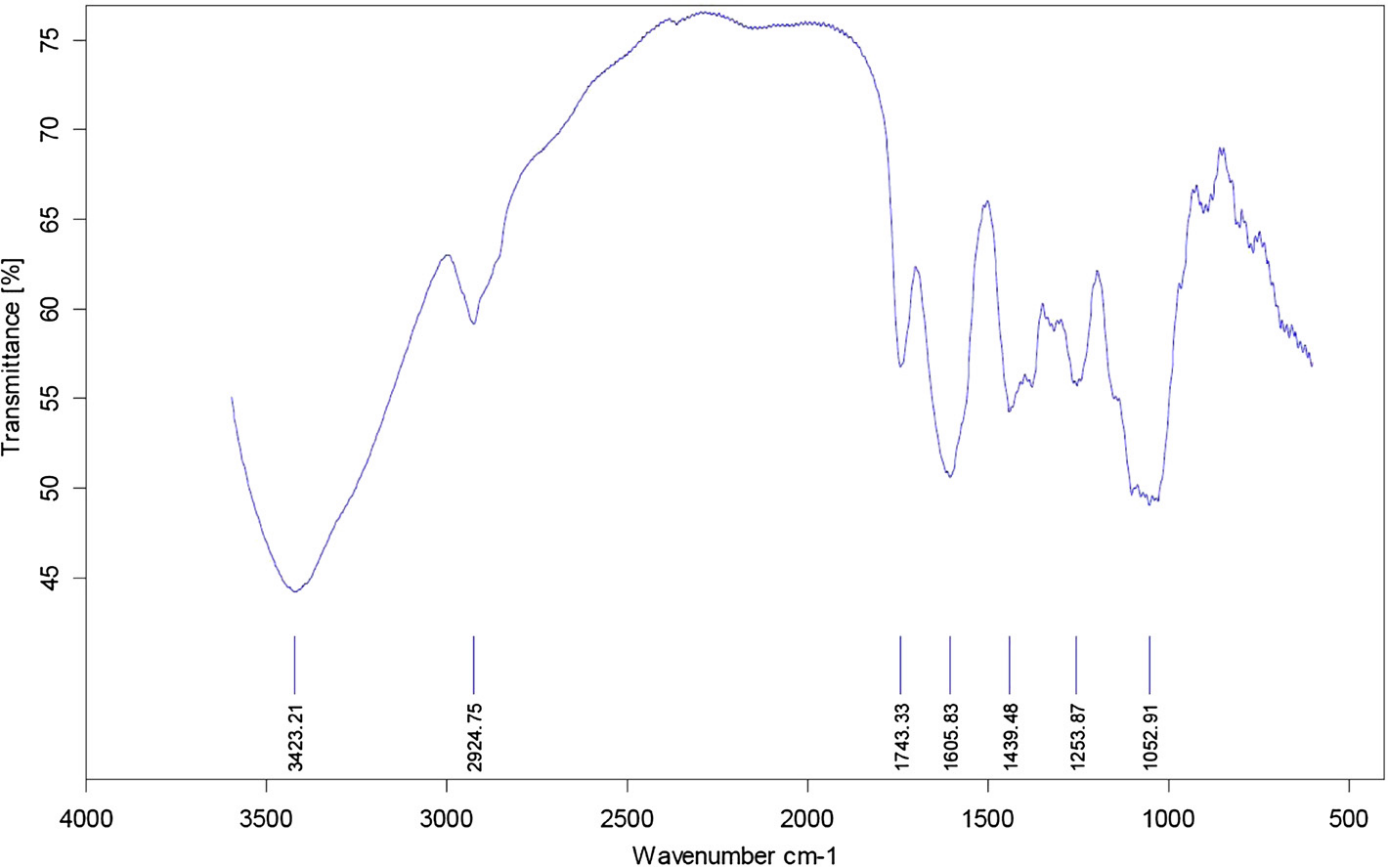
**The FT-IR spectra of the crude polysaccharides of *A. vera *in T150 NPK fertilizer treatment.**

Different combinations of fertilizers significantly affected the crude polysaccharides of *A. vera* in this experiment (Figures 3, 4, 5, and 6). The highest content of the crude polysaccharides of *A. vera* was observed from T_4_ (50% N + 50% P + 50% K) followed by T_3_ (25% N + 25% P + 100% K). Concerning total carbohydrate content, the data in Figures 3, 4, 5, and 6 indicate that all treatments significantly increased the carbohydrate content as compared to the control plant.

Using (50% N + 50% P + 50% K) fertilizer was the most effective in increasing the crude polysaccharide content of *A. vera* (75%). The effect of T150 N was not superior to the fertilizer in this experiment.

## Conclusions

In the process of preparing *A. vera*, the leaf skin is usually disposed of as waste, and aloe flower is also underestimated due to the small amount of origin from *A. vera*. The results from this work indicate that a considerable amount of polysaccharides is present in the skin juice, which is 2.4 times the level in gel juice; thus, we suggest that *A. vera* skin should be used fully for the extraction of polysaccharides rather than discarded. *A. vera* flower also contains an abundant amount of polysaccharides. However, the concentration of carbohydrates detected in the first ethanol-precipitated solid from the skin juice was rather low. This could be owing to the presence of co-precipitated protein and possible oxalate materials. Also, the polysaccharide solution extracted from aloe flower was more contaminated with protein and colored materials or phenolic compounds; thus, additional purification steps were necessary to remove these materials. Neutral polysaccharides accounted for 59.1% and 79.8% of the total recovered polysaccharide preparations for the skin and gel juices, respectively, with small amounts of acidic polysaccharides obtained after DEAE-Sephadex A-25 anion-exchange chromatography. In contrast, the polysaccharides of *A. vera* flower were mainly composed of acidic polysaccharide (85.2%). After further purification by gel permeation chromatography, component sugar analysis by gas–liquid chromatography indicated that there were significant differences between the neutral polysaccharides and acidic polysaccharides from different tissue zones of *A. vera*. In the neutral polysaccharides from the gel juice and flower tissues, glucose was the largest component, which accounted for around 81% and 40%, respectively. Also, the neutral polysaccharide from the skin juice was composed mostly of galactose (62.8%). Additionally, in the acidic polysaccharides detected in the experiment from the gel juice and flower tissues, galactose and glucose were the major monosacccharides, while the acidic polysaccharide from the skin tissue was mainly consisted of glucose and mannose. These results were broadly similar to those obtained for the gel polysaccharides of *A. vera* by Hikino et al. [5]; on the contrary, Wozniewski et al. [7] found that mannose was the predominant sugar. Glucuronic acid was the major uronic acid in all acidic polysaccharides from different tissues.

This trend of increased polysaccharides due to increased application of fertilizer was also observed in the case of the total weight of leaves, aloe gel, and skin juice. The application of organic fertilizer increased the total weight of leaves and crude polysaccharides without hampering the nutrient uptake process, which provided better results due to better medicinal value. Further studies of the structure and biological role of these polysaccharides are necessary, and parts of them are in progress in our group.

## Competing interests

The authors declare that they have no competing interests.
